# Epithelial–mesenchymal transition, IP_3_ receptors and ER–PM junctions: translocation of Ca^2+^ signalling complexes and regulation of migration

**DOI:** 10.1042/BJ20150364

**Published:** 2016-03-10

**Authors:** Emmanuel Okeke, Tony Parker, Hayley Dingsdale, Matthew Concannon, Muhammad Awais, Svetlana Voronina, Jordi Molgó, Malcolm Begg, Daniel Metcalf, Alex E. Knight, Robert Sutton, Lee Haynes, Alexei V. Tepikin

**Affiliations:** * Department of Cellular and Molecular Physiology, University of Liverpool, Crown Street, Liverpool L69 3BX, U.K.; † NIHR Liverpool Pancreas Biomedical Research Unit, University of Liverpool, Crown Street, Liverpool L69 3BX, U.K.; ‡ CEA, Institut de Biologie et Technologies de Saclay (iBiTec-S), Service d'Ingénierie Moléculaire des Protéines (SIMOPRO), bâtiment 152, 91191 Gif-sur-Yvette Cedex, France; § Institut des Neurosciences Paris-Saclay, UMR 9197, CNRS/Université Paris-Sud, CNRS, 91190-Gif sur Yvette Cedex, France; ║ Respiratory Therapy Area Unit, Medicines Research Centre, GlaxoSmithKline, Stevenage SG1 2NY, England, U.K.; ¶ Biotechnology Group, National Physical Laboratory, Hampton Road, Teddington TW11 0LW, U.K.

**Keywords:** epithelial–mesenchymal transition, ER–PM junctions, focal adhesions, IP_3_ receptors, pancreatic ductal adenocarcinoma, PANC-1 cells

## Abstract

Disconnection of a cell from its epithelial neighbours and the formation of a mesenchymal phenotype are associated with profound changes in the distribution of cellular components and the formation of new cellular polarity. We observed a dramatic redistribution of inositol trisphosphate receptors (IP_3_Rs) and stromal interaction molecule 1 (STIM1)-competent endoplasmic reticulum–plasma membrane junctions (ER–PM junctions) when pancreatic ductal adenocarcinoma (PDAC) cells disconnect from their neighbours and undergo individual migration. In cellular monolayers IP_3_Rs are juxtaposed with tight junctions. When individual cells migrate away from their neighbours IP_3_Rs preferentially accumulate at the leading edge where they surround focal adhesions. Uncaging of inositol trisphosphate (IP_3_) resulted in prominent accumulation of paxillin in focal adhesions, highlighting important functional implications of the observed novel structural relationships. ER–PM junctions and STIM1 proteins also migrate to the leading edge and position closely behind the IP_3_Rs, creating a stratified distribution of Ca^2+^ signalling complexes in this region. Importantly, migration of PDAC cells was strongly suppressed by selective inhibition of IP_3_Rs and store-operated Ca^2+^ entry (SOCE), indicating that these mechanisms are functionally required for migration.

## Abstract (precis)

During epithelial–mesenchymal transition IP_3_Rs relocate from tight junctions to the leading edge of migrating pancreatic cancer cells and regulate dynamics of focal adhesions. STIM1-competent ER–PM junctions position closely behind IP_3_Rs and, together with IP_3_Rs, regulate cell migration.

## INTRODUCTION

Pancreatic ductal adenocarcinoma (PDAC) is a leading cause of cancer-related death [1]. Epithelial–mesenchymal transition (EMT), migration and invasion are cellular processes that are crucially important for the formation of lethal metastases in this and other types of cancer (reviewed in [2–4]). Currently, understanding the fundamental contributions that Ca^2+^ signalling makes to cell migration is an important research avenue of potential clinical relevance [5–7]. It is particularly relevant for identifying putative therapeutic targets that could delay or prevent the formation of metastases (reviewed in [8]). Two prominent components of the Ca^2+^ signalling cascade are inositol trisphosphate receptors (IP_3_Rs) and the store-operated Ca^2+^ entry (SOCE) mechanism. IP_3_Rs are intracellular Ca^2+^-releasing channels [9] which mediate responses to numerous hormones and neurotransmitters (reviewed in [10,11]). SOCE restores the Ca^2+^ concentration in the ER ([Ca^2+^]_ER_) following its depletion due to the Ca^2+^-releasing activity of IP_3_Rs or other intracellular channels (reviewed in [12,13]). The endoplasmic reticulum–plasma membrane junctions (ER–PM junctions) are regions of close contact between the two organelles [14–16] which serve as hubs for cAMP [17,18], phospholipid [19] and Ca^2+^ [16,20,21] signalling. SOCE is a Ca^2+^ influx mechanism triggered by Ca^2+^ store depletion, which involves the oligomerization of stromal interaction molecule 1 (STIM1, an EF-hand-containing protein that serves as the ER Ca^2+^ sensor), translocation of STIM1 oligomers to ER–PM junctions and opening of PM Ca^2+^ channels [16,22–25]. STIM1-competent ER–PM junctions can thus be classed as platforms for SOCE. Orai proteins are pore-forming components of SOCE channels that are opened by STIM1 [22,24,26], although some types of transient receptor potential (TRP) channels are probably also involved [27]. Following [Ca^2+^]_ER_ depletion, Orai proteins translocate to the PM component of the ER–PM junctions where they interact with STIM and form Ca^2+^-selective channels. Ca^2+^ is heavily buffered in the cytosol of most cell types (e.g. [28,29]) and therefore proximity of Ca^2+^ channels to their downstream targets is frequently crucial for the efficiency and specificity of signalling. In the case of migrating cells and the regulation of migration, the relative positioning of Ca^2+^ signalling complexes, proteins that define the leading edge and focal adhesions (structures responsible for interaction between the cell and extracellular matrix), is of particular interest.

In the present study we have characterized the drastic redistribution of Ca^2+^ signalling complexes and ER–PM junctions occurring when PDAC cells disconnect from their neighbours and develop a migratory phenotype. Furthermore, we have characterized novel stratified localization of the Ca^2+^ signalling complexes in the leading edge and their structural relationships with the components of migratory apparatus. Finally, we have revealed the functional importance of IP_3_Rs and of SOCE for cell migration.

## MATERIALS AND METHODS

### Reagents

Xestospongin-B was provided by Dr J. Molgó (Institut de Biologie et Technologies de Saclay, Saclay, France) and GSK-7975A was a gift from Dr Malcolm Begg (GlaxoSmithKline, Stevenage, U.K.). Cyclopiazonic acid (CPA) was from Tocris, thapsigargin (TG) and rapamycin were purchased from Calbiochem, caged Ins(1,4,5)*P*_3_/PM (cag-iso-2-145) was purchased from Sichem. RIPA lysis and extraction buffer, Halt protease inhibitor cocktail and EDTA supplements were purchased from Pierce-Thermo Scientific. Fura-2-acetoxymethyl ester (AM), Fluo-4-AM, 1,2-bis-(*o*-aminophenoxy)ethane-*N,N,N′,N′*-tetra-acetic acid (BAPTA)-AM, Hoechst 33342, Sytox Orange, propidium iodide, human IP_3_R siRNA oligomers, anti-GFP (rabbit and chicken), fluorophore-conjugated antibodies and Alexa Fluor 647 phalloidin were all purchased from Life Technologies.

### Primary antibodies

Anti-IP_3_R1 [rabbit polyclonal raised against C-terminal amino acids 2735–2749 [30] was a gift from Professor J. Parys (Catholic University of Leuven, Leuven, Belgium)]. Anti-IP_3_R1 [D53A5 (rabbit)] was from Cell Signaling Technology. Anti-IP_3_R2 (rabbit polyclonal raised against C-terminal amino acids 2686–2702) was a gift from Professor D. Yule (University of Rochester, Rochester, NY, U.S.A.); anti-IP_3_R3 (mouse) was from BD Transduction Laboratories. Anti-pan-IP_3_R rabbit polyclonal raised against C-terminal epitopes was from Millipore. Anti-occludin (rabbit and mouse) was from Zymed Laboratories. Anti-vinculin (mouse), anti-β-actin (mouse) and horseradish peroxidase (HRP)-conjugated secondary antibodies (anti-rabbit and anti-mouse species) were from Sigma–Aldrich. Anti-E-cadherin (mouse) was from Santa Cruz Biotechnology.

### Cell culture, constructs and transfection

PDAC cell line PANC-1 was obtained from the ATCC (ATCC number CRL-1469) and cultured in DMEM (Dulbecco's modified Eagle's medium) supplemented with 10% FBS (fetal bovine serum), 100 units/ml penicillin, 100 μg/ml streptomycin and 292 μg/ml glutamine. Cultured cells were maintained in a humidified incubator (Wolf Laboratories) at 37°C and 5% CO_2_.

DNA constructs coding for LL–FKBP–mRFP (where LL denotes a long linker, FKBP is FK506-binding protein and mRFP is monomeric RFP), CFP–FRB–LL (FRB is FKBP12–rapamycin-binding) [21] and for YFP–STIM1 with a TK (thymidine kinase) promoter [21] were gifts from Dr T. Balla (National Institute of Child Health and Human Development, Bethesda, MD, U.S.A.).

In our study we utilized mCherry-labelled paxillin (Pax-mCh) and paxillin labelled with the Ca^2+^ sensor GCaMP5 (Pax-GCaMP5). The coding sequence for GCaMP5 was obtained from Addgene (plasmid 31788, originally generated by Douglas Kim and Loren Looger [31]). The GCaMP5 coding sequence was PCR-amplified using the following primer pair containing restriction endonuclease sites (underlined) to permit sub-cloning into the pcDNA3.1(+) backbone (Life Technologies) generating a new ‘pcDNA-GCaMP5-CT’ C-terminally tagging expression vector: sense (NotI) 5′-ATATGCGGCCGCATG-ACTGGTGGACAGCAAATG-3′; antisense (ApaI) 5′-ATA-TGGGCCCTCACTTCGCTGTCATCATTTGTAC-3′. Pax-GCaMP5 was created by PCR amplification of the chicken paxillin sequence obtained from Addgene (plasmid 15233, originally deposited by Rick Horwitz [32]) using the following primer pair containing restriction endonuclease sites (underlined) to permit subcloning into pcDNA-GCaMP5-CT: sense (EcoRI) 5′-ATATGAATTCACCATGGACGACCTC-GATGCC-3′; antisense (NotI) 5′-ATATGCGGCCGCTAC-AGAAGAGTTTGAGAAAGC-3′. Pax-mCh was created by PCR amplification of the chicken paxillin sequence using the following primer pair containing restriction endonuclease sites (underlined) for subcloning into the mCherry-N1 vector (Clontech): sense (XhoI) 5′-ATATCTCGAGACCATGGACG-ACCTCGATGCC-3′; antisense (EcoRI) 5′-ATATGAATTC-GACAGAAGAGTTTGAGAAAGCA-3′. All constructs were verified by automated sequencing (The Sequencing Service, University of Dundee, Dundee, U.K.).

To express the proteins of interest, cells were transfected at approximately 60–70% confluence with 1–2 μg of DNA per plasmid construct for 24 h using PromoFectin reagent (PromoKine) according to the manufacturer's instructions. For the knockdown of cellular proteins of interest, siRNA oligomers directed against human IP_3_R1, IP_3_R2 and IP_3_R3 isoforms were used. Cells were transfected at approximately 30–40% confluence with 50 nM per siRNA oligomer for 72 h using Lipofectamine 2000 (Life Technologies) according to the manufacturer's instructions.

### Immunofluorescence and visualising ER–PM junctions

Cells were seeded on to 35-mm-diameter glass-bottom dishes from Mattek or ibidi.

For visualising ER–PM junctions, cells were transfected with both PM-targeted LL–FKBP–mRFP and ER-targeted CFP–FRB–LL constructs, and 24 h later, were treated with 100 nM rapamycin for 4–5 min at 37°C/5% CO_2_. For further details, see [21,33].

For visualising STIM1 puncta, cells were transfected with YFP–TK–STIM1, and 24 h later, were treated with 30 μM CPA for 1 h at 37°C/5% CO_2_ and imaged using a confocal microscope.

For immunostaining, cells were fixed using 4% (v/v) paraformaldehyde (PFA) in PBS for 10–15 min at room temperature (approximately 18–22°C) followed by three PBS washes, and subsequently permeabilized using 0.2% Triton X-100 in PBS for 5 min at room temperature, before an additional three PBS washes. Non-specific antibody binding was blocked for 1 h at room temperature in PBS containing 10% (v/v) goat serum and 1% (w/v) BSA prior to incubation with primary antibodies in PBS containing 5% (v/v) goat serum and 0.1% acetylated BSA for 1 h at room temperature or overnight at 4°C. Primary antibodies were used at the following dilutions: anti-IP_3_R1, 1:200; anti-IP_3_R2, 1:100; anti-pan-IP_3_R, 1:20; anti-vinculin, 1:200; anti-GFP, 1:200; anti-occludin, 1:100; anti-E-cadherin, 1:50. After the incubation with primary antibodies, cells were washed with PBS three times followed by the addition of appropriate species-specific fluorophore-conjugated secondary antibodies for 30 min at room temperature at dilutions of 1:500–1:1000 in PBS. Additional three PBS washes were carried out prior to imaging in PBS. In the specified experiments, Alexa Fluor 647 phalloidin was used (at 1:50 dilution).

Two different confocal microscopes were used to visualize the distribution of specific proteins and ER–PM junctions in fixed cells: Leica TCS SP2 (AOBS) confocal microscope with ×63 oil-immersion objective [NA (numerical aperture) − 1.4] and Zeiss LSM 710 confocal microscope with ×63 oil-immersion objective (NA − 1.4). The pinhole was set between 1 and 2 airy units.

### Live-cell Ca^2+^ imaging and uncaging

To investigate inositol trisphosphate (IP_3_)-induced Ca^2+^ responses, cells were loaded with caged IP_3_ and with Ca^2+^ indicator Fluo-4 by incubation in the solution containing 1 μM caged IP_3_/PM and 5 μM Fluo-4-AM. A Zeiss LSM 510 confocal microscope with a ×63 water-immersion objective (NA 1.2) was utilized in these experiments; the 488 nm laser line was used to excite Fluo-4 (emission recorded at LP 505 nm), 351 nm and 364 nm laser lines were used for uncaging.

To investigate SOCE, PANC-1 cells were loaded with Fura-2 by incubation for 1 h in Fura-2-AM-containing solution. Cells were washed for 30 min to allow de-esterification of the probe. A Till Photonics Imaging system was used in these experiments. Fluorescence of cells loaded with Fura-2 was excited at 340 and 380 nm, and emission collected using a 510 nm bandpass filter. Data recorded in these experiments are expressed as the ratio of fluorescence excited by 340 nm (*F*340) and 380 nm (*F*380), after corresponding background subtraction. Changes of extracellular solution were made using gravity-fed perfusion system.

### Migration assay

Boyden chambers were purchased from Corning. The pore size was 8 μm. Migration was measured in conditions of symmetrical FBS (1% FBS in both the upper and lower chambers) and asymmetrical FBS (0% FBS in the upper chamber and 5% FBS in the lower chamber). After seeding, PANC-1 cells were allowed to migrate for 6 h in a humidified environment at 37°C/5% CO_2_ in the presence or absence of the inhibitors of various components of the Ca^2+^ signalling cascade; Boyden chamber inserts were then fixed using 100% methanol and non-migrated cells were removed from the top-side of the inserts using cotton buds. The inserts were then rinsed two times in PBS, prior to the staining of migrated cells on the underside of the chamber inserts using 100 μg/ml propidium iodide. The inserts containing fixed and stained cells were imaged on a Leica AOBS TCS SP2 confocal microscope using a ×10 air objective with an NA of 0.3 as described in [34]. Five representative regions of interest were imaged per insert. Fluorescently stained migrated cells were counted using CellProfiler software cell counting algorithm.

### Super-resolution imaging

For super-resolution imaging, PANC-1 cells were seeded into Lab-Tek chambered coverglass eight-well #1.0 with low thickness variation (Thermo Scientific). To visualize IP_3_R1, PANC-1 cells were fixed with 4% PFA for 10–15 min at room temperature and subsequently immunostained with anti-IP_3_R1 antibody, followed by the use of an appropriate species-specific Alexa Fluor 647-conjugated secondary antibody. To visualize ER–PM junctions at super-resolution level, PANC-1 cells co-transfected with PM-targeted LL–FKBP–mRFP and ER-targeted CFP–FRB–LL constructs for 24 h were fixed using 4% PFA for 10–15 min at room temperature after treatment with 100 nM rapamycin for 4–5 min at 37°C/5% CO_2_ to highlight the pre-existing ER–PM junctions without ER Ca^2+^ store depletion. Briefly, the heterodimerization of both ER- and PM-targeted constructs revealed the ER–PM junctions as punctate structures in both CFP and RFP fluorescence channels. To highlight the ER-targeted FRB–LL–CFP counterpart of the ER–PM junctions' puncta, cells were immunostained using anti-GFP antibody (which also recognizes CFP), and followed by appropriate species-specific Alexa Fluor 647-conjugated secondary antibody.

After immunostaining of IP_3_R1 or ER–PM junctions, samples were immersed and imaged in a dSTORM buffer containing 100 units/ml glucose oxidase, 2000 units/ml catalase, 50 mM mercaptoethylamine/HCl and 50 mg/ml glucose in PBS [35]. Wells were filled and sealed with a coverslip to exclude oxygen. Super-resolution microscopy was performed using a custom-built instrument, as described previously [35,36]. An Olympus IX71 microscope formed the basis of an inverted objective total internal reflection fluorescence (TIRF) instrument with a UAPON ×100 TIRF, NA − 1.49 objective. Laser illumination was provided by a 640 nm 150 mW diode laser (Toptica Photonic AG) and a 561 nm 200 mW optically pumped semiconductor laser (Coherent Europe). Additionally, a 405 nm laser diode (Mitsubishi Electronics) was available for re-activation of fluorophores if required. Laser power on the diode lasers was controlled directly, whereas a rotating quarter-wave plate was used to alter the power of the 561 nm laser. Low powers (1–10%) were used for field of view selection, context and conventional fluorescence images. Higher powers (50–100%) were used for dSTORM imaging. Fluorescence and excitation light were spectrally separated by the dichroic mirror and emission filter from a multi-edge filter set (LF405/488/561/635-A-000, Semrock), and an additional bandpass filter was used to remove cross-talk in each channel (FF01-676/37 with 640 nm excitation and FF01-600/37 with 561 nm excitation, both from Semrock).

Images were acquired using an EMCCD camera (Andor iXon 897) using software written in LabVIEW (National Instruments). Typically dSTORM image ‘stacks’ were composed of 10000 frames, with 10 ms exposure time per frame. Super-resolution images were reconstructed from these image stacks using the open-source rainSTORM package [35,37,38].

### IP_3_Rs knockdown and cell migration

Migration of PANC-1 cells after 72 h of siRNA knockdown of IP_3_R1 or IP_3_R2 or IP_3_R3 isoforms was measured using a Boyden chamber assay. siRNA sequences targeting each of the IP_3_R isoforms are: IP_3_R1 silencer select siRNA sense 5′-GCACGACAGUGAAAACGCAtt-3′, antisense 5′-UGCGUUUUCACUGUCGUGCct-3′; IP_3_R2 silencer select siRNA sense 5′-GGUGUCUAAUCAAGACGUAtt-3′, antisense 5′-UACGUCUUGAUUAGACACCag-3′; and IP_3_R3 silencer select siRNA sense 5′-GCAUGGAGCAGAUCGUGUUtt-3′, antisense 5′-AACACGAUCUGCUCCAUGCtg-3′. Following 72 h of treatment with the specified siRNA constructs, PANC-1 cells were allowed to migrate for 6 h in a humidified environment at 37°C/5% CO_2_. Boyden chamber inserts were then fixed using 100% methanol. Non-migrated cells were removed from the top-side of the chamber inserts using cotton buds and rinsed two times in PBS, prior to the staining and counting of migrated cells on the underside of the chamber inserts (see the Migration Assay section above for the description of staining and counting procedures).

### Immunoblotting

Cells were treated with trypsin, removed from flasks and then collected by centrifugation. Cells were then lysed using RIPA lysis extraction buffer supplemented with Halt protease inhibitor cocktail and EDTA (Pierce-Thermo Scientific). Lysed samples were separated on a 4–12% NuPAGE Bis–Tris gradient gels and protein transferred on to nitrocellulose membranes by transverse electrophoresis. Nitrocellulose membranes were blocked in 3% (w/v) non-fat dried skimmed milk powder dissolved in PBS for 1 h at room temperature, and probed with primary antibodies including anti-IP_3_R1 (1:500 dilution), anti-IP_3_R2 (1:1000 dilution), anti-IP_3_R3 (1:500 dilution) and anti-β-actin (1:1000 dilution) at 4°C overnight. After overnight incubation, nitrocellulose membranes were incubated with appropriate species-specific HRP-conjugated secondary antibodies (1:400 dilution) for 1 h at room temperature. Bands were visualized using enhanced chemiluminescence (ECL) Western blotting substrate and a Bio-Rad Quantity One imaging system. Band intensities were quantified and analysed using ImageJ software (NIH). Blotting for β-actin was used as a loading control for siRNA knockdown experiments.

### Image, data and statistical analyses

Image acquisition and preliminary analysis of confocal images was performed using either Leica LAS or Zeiss LSM 510 or Zeiss Zen software. Further analysis was performed using ImageJ software. Linear adjustments of contrast and brightness were applied if necessary using ImageJ. The ‘mask’ images used for illustrating the co-localization of the rapamycin-inducible linker components (images labelled ER–PM linkers) were created using the Co-localize RGB ImageJ plugin as described in [33].

In data presentation (for all components of the study) the error margins represent the S.E.M. The results were analysed using a Student's *t* test; *P*<0.05 was considered statistically significant and is indicated by the symbol * in the Figures.

## RESULTS AND DISCUSSION

### IP_3_Rs translocate from cell–cell contacts in cellular clusters to the leading edge of individual migrating cancer cells

**Figure 1:**
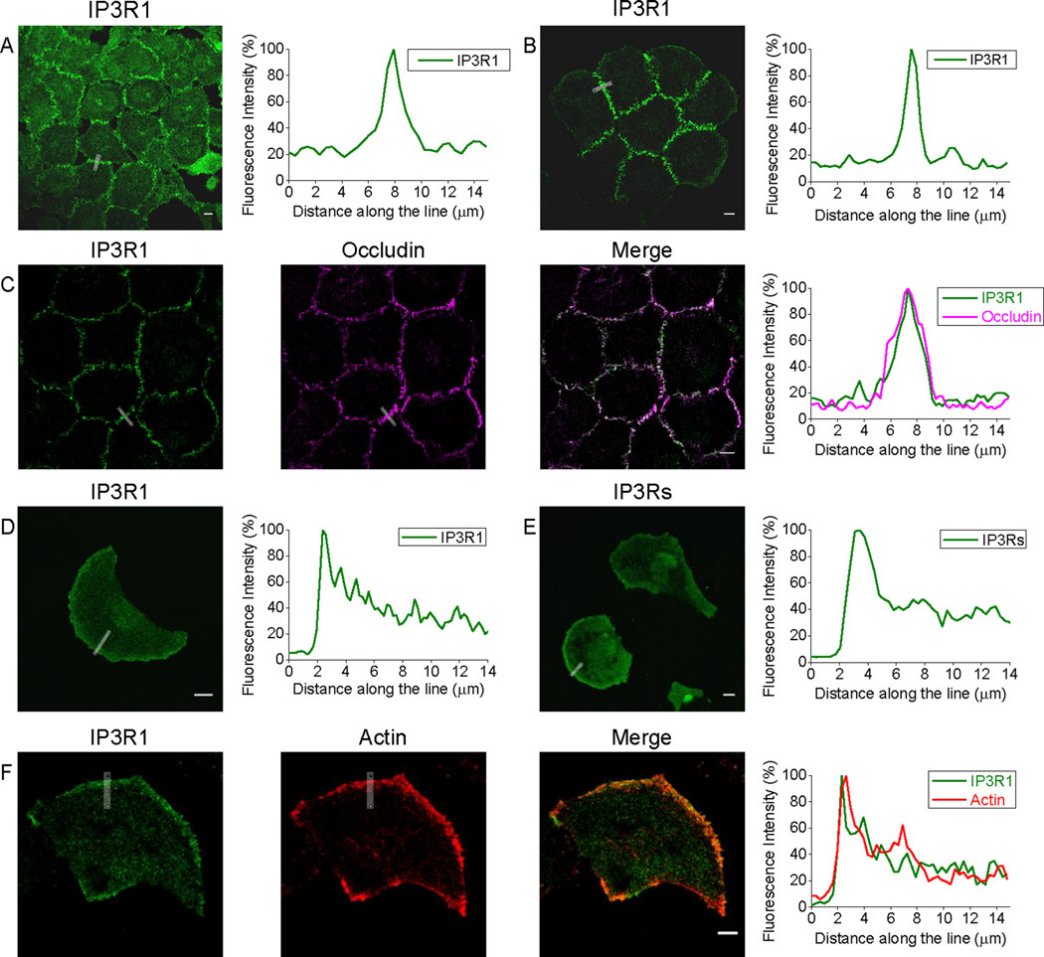
The polarized distribution of IP_3_R1s in connected PANC-1 cells and in isolated migrating PANC-1 cells (**A**) IP_3_R1s are localized at the cell–cell contact sites of PANC-1 cells forming a confluent monolayer. (**B**) IP_3_R1s are also localized at the cell–cell contact sites of small clusters of PANC-1 cells. (**C**) IP_3_R1s co-localize with occludin (tight junction marker) at the cell–cell contact sites of PANC-1 cells. Fluorescence profiles (right panel) that illustrate juxtaposition of the proteins were measured along the line spanning the cell-cell contact region. (**D**) IP_3_R1 predominantly decorates the leading edge of a migrating PANC-1 cell (immunostained using anti-IP_3_R1 antibody). (**E**) IP_3_Rs predominantly decorate the leading edge of PANC-1 cell (immunostained using pan-IP_3_R antibody). (**F**) IP_3_R1 co-positions with actin-rich lamellipodia at the leading edge of migrating pancreatic adenocarcinoma cells. Other examples of specific localization of IP_3_Rs are shown in Supplementary Figures S1–S3. Scale bars represent 10 μm.

In monolayers of PANC-1 cells, IP_3_R1 was observed primarily in the areas of cell–cell contacts (see Figure 1A and Supplementary Figure S1). Similar distribution was observed in smaller cellular clusters (Figure 1B). Fluorescence profiles measured along the lines selected to cross junctional regions demonstrate approximately 4–5-fold increased density of IP_3_R1 in these regions in comparison with the neighbouring regions of the cytoplasm (Figures 1A and 1B, and Supplementary Figures S1A and S1C, upper panel). In cell–cell contact regions IP_3_R1 was closely co-positioned with occludin, which we used as a marker of tight junctions (Figure 1C). The white colour in the ‘Merge’ panel of this figure indicates that in a part of this region the IP_3_R1 and occludin are in such close proximity that the distance between these proteins is below the resolution of a confocal microscope (i.e. less than 300 nm). Notably, in the monolayers of PANC-1 cells, E-cadherin (a component of adherence junctions and an important marker of the epithelial phenotype) was also preferentially found in the cell-cell contact regions (Supplementary Figure S2), confirming cellular connectivity.

The observed proximity of IP_3_Rs to tight junctions was described before in non-transformed Madin–Darby canine kidney (MDCK) cells, where it was associated with the developed epithelial phenotype [30,39]. Interestingly, in our experiments this distribution was observed in the monolayers of cancer cells (PANC-1 cells) and even in relatively small clusters of these cells. Importantly, cells disconnected from the clusters or monolayers also clearly display polarized distribution of IP_3_R1, as these cells preferentially positioned their IP_3_R1s at the leading edge (Figure 1D, and Supplementary Figures S1A, right panels, and S1C, central panel). Notably, similar increased density of IP_3_R1 at the leading edge was observed in migrating PANC-1 cells after treatment with transforming growth factor (TGF)-β1, an agonist known to induce a migratory mesenchymal phenotype in this cell type [40,41] (see Supplementary Figure S3). Preferential positioning at the leading edge was also observed for IP_3_R2s (Supplementary Figure S4). Pan-IP_3_R antibodies, which were raised against a conserved C-terminal region common to all IP_3_R subtypes, also revealed a similarly polarized distribution of IP_3_Rs with preferential localization at the leading edge of individual migrating cells (Figure 1E and Supplementary Figure S1C, lower panel). The leading edge of migrating cells is characterized by the region of polymerized actin (reviewed in [4]). We therefore examined the relative positioning of the F-actin-enriched region and IP_3_R1; Figure 1(F) illustrates close apposition of the two proteins at the leading edge of isolated migrating PANC-1 cells. In addition to actin polymerization, cellular migration requires the formation of focal adhesions at the front of migrating cells; we therefore extended our analyses to examine the relative positioning of IP_3_Rs and focal adhesions.

### Focal adhesions are closely surrounded and regulated by IP_3_Rs at the leading edge of migrating cancer cells

**Figure 2:**
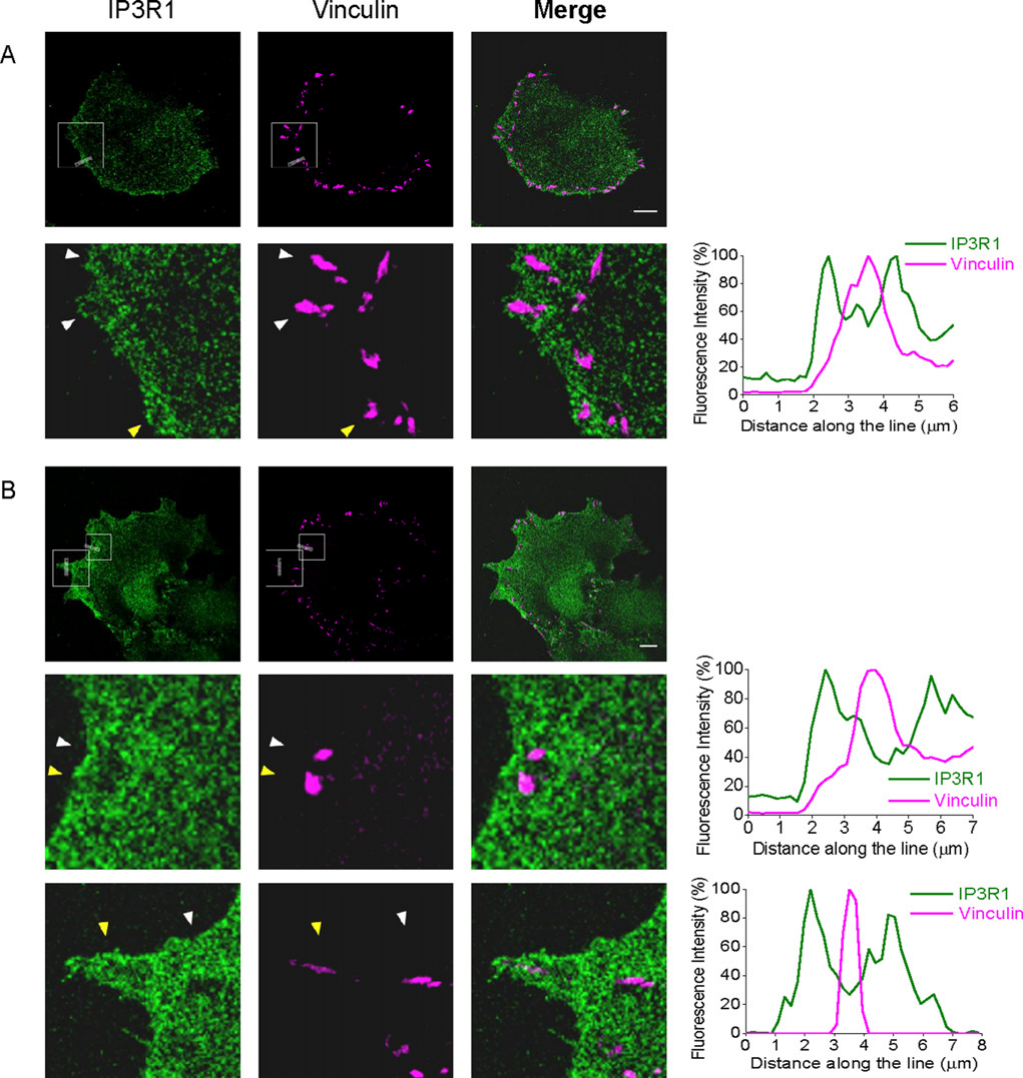
IP_3_Rs encompass focal adhesions in migrating PANC-1 cells Non-transfected PANC-1 cells were fixed and stained with anti-IP_3_R1 and anti-vinculin antibodies. Scale bars represent 10 μm. (**A**) IP_3_R1s encompass focal adhesions in migrating pancreatic adenocarcinoma cells with a smooth leading edge. An expanded fragment of the cell is shown in the lower panels and the arrowheads indicate where the focal adhesions are encompassed by IP_3_R1s. Fluorescence profile (right panel) was measured along the line (shown in the top left and top central images) spanning the cell membrane of the leading edge and crossing a focal adhesion (specifically the lowest of three focal adhesions indicated by the yellow arrowhead in the expanded fragment). (**B**) The relationships between IP_3_R1s and focal adhesions in a cell with a complex leading edge composed of smooth (lamellipodia-like) regions and some spiky protrusions. The central panels correspond to the upper box in the cell image. These panels and the associated fluorescence profile represent an expanded fragment of a smooth region (similar to **A**), whereas the bottom panels show a representative spiky protrusion. In both cases the focal adhesions (indicated by arrowheads) are surrounded by IP_3_R1s. The associated fluorescence profiles were measured across the lines shown in the upper panels; the focal adhesions crossed by these lines are indicated by yellow arrowheads on central and lower panels.

Dual immunostaining of focal adhesions (using antibodies against vinculin) and IP_3_R1s revealed remarkable relative localization of the adhesions and the receptors. As expected, focal adhesions were preferentially localized close to the leading edge of the migrating cells; the leading edge was also enriched with IP_3_R1s (Figure 2A). Importantly, focal adhesions were not co-localized but were instead closely surrounded by the receptors forming ‘potholes’ on the background of IP_3_R1 immunostaining (see the fragment shown in the bottom panels in Figure 2(A) and the associated fluorescence profile). The preferential positioning of focal adhesions and IP_3_Rs as well as ‘potholes’ were observed in the smooth-shaped (lamellipodia-like) regions of the leading edge (Figure 2A and the central row of images in Figure 2B with the associated fluorescence profile) as well as in spiky, filopodia-like protrusions at the leading edge (Figure 2B and specifically the lower row of panels and the associated fluorescence profile). The intimate spatial relationship between focal adhesions and IP_3_Rs concentrated in this region should make these structures particularly sensitive to Ca^2+^ signals generated by IP_3_Rs at the leading edge of migrating cells and could explain the prominent effect of the inhibition of IP_3_Rs on cell migration (see the last subsection of the Results section). Using intracellular photorelease of IP_3_ from its caged precursor (uncaging) we directly probed the influence of this second messenger on the dynamics of focal adhesions. In these experiments we used cells expressing simultaneously Pax-mCh and Pax-GCaMP5. Paxillin was used in these experiments because it is an important regulatory component of focal adhesions [42].

**Figure 3:**
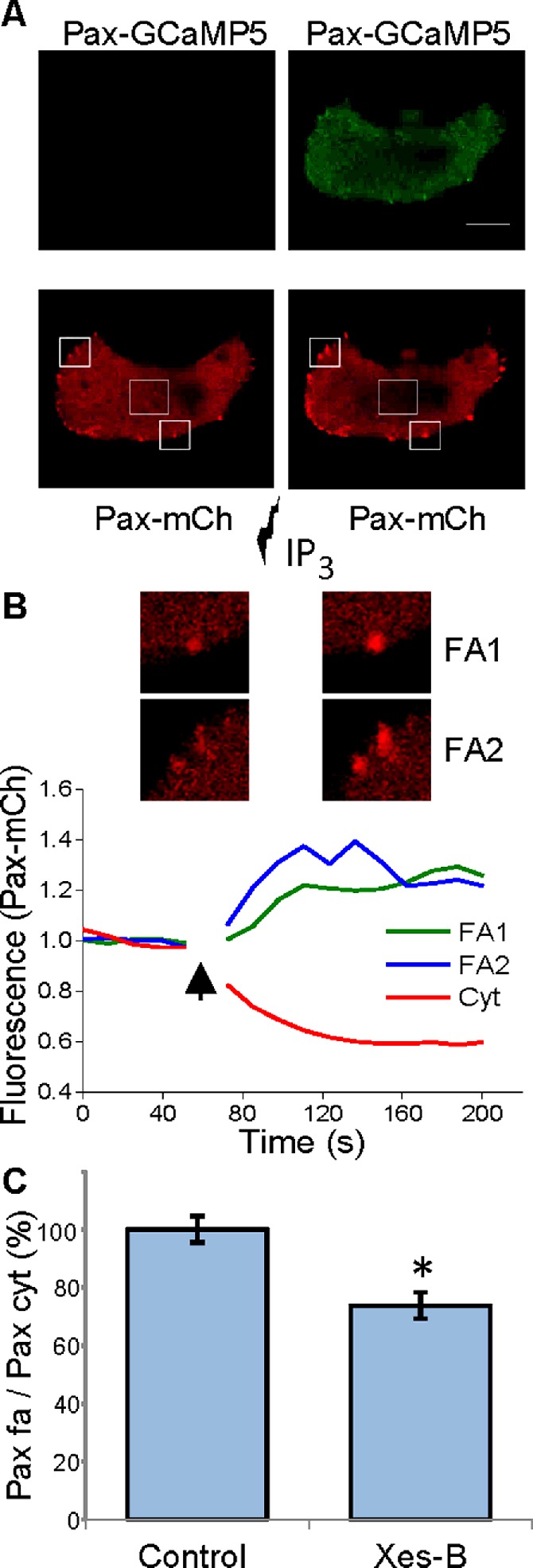
IP_3_Rs and remodelling of focal adhesions in PANC-1 cells (**A**) IP_3_ uncaging induces paxillin accumulation in focal adhesions. Upper panels show the fluorescence of Pax-GCaMP5 before (left) and after (right) uncaging. The increase in GCaMP5 fluorescence indicates a Ca^2+^ rise; note the prominent response in focal adhesions. The lower panels show the distribution of Pax-mCh fluorescence before (left) and after (right) IP_3_ uncaging. Note the prominent increase in fluorescence in focal adhesions induced by the uncaging. (**B**) The graph shows the increase in fluorescence (i.e. Pax-mCh accumulation), recorded from the regions of interest containing focal adhesions (shown above the traces and highlighted by solid border boxes in **A**), and the decrease in Pax-mCh fluorescence in the cytosol (the region of interest for this analysis included the area of cytosol highlighted by dashed border box in **A**). The arrow indicates the period of uncaging (during this period the fluorescence recording was interrupted). The images are fragments of the cell shown in (**A**). (**C**) Xestospongin-B (Xes-B) produced a statistically significant decrease in paxillin content in the focal adhesions of unstimulated cells (i.e. cell are not treated with IP_3_-generating agonists). In these experiments cells were stained with anti-paxillin antibodies. The fluorescence of focal adhesions was normalized by the fluorescence of the cytosol. The normalized fluorescence of paxillin in focal adhesions was measured in untreated cells (control, *n*=39) and in cells incubated with xestospongin-B for 1 h (*n*=32). The results for both groups are shown as mean values ± S.E.M.

Uncaging of IP_3_-induced rapid accumulation of paxillin in focal adhesions and the loss of paxillin from the cytosol (Figures 3A and 3B). These findings highlight the importance of IP_3_Rs for focal adhesion remodelling–the process immediately related to migration ([43], reviewed in [42]). Uncaging of IP_3_ also induced Ca^2+^ rises resolvable both in the cytosol and localized at focal adhesions (Figure 3A). Interestingly, inhibition of IP_3_Rs with xestospongin-B reduced the paxillin content of focal adhesions in unstimulated PANC-1 cells (Figure 3C), suggesting that IP_3_Rs are involved in the regulation of focal adhesion even when cells are not stimulated with IP_3_-producing agonists. These results suggest that focal adhesions are modulated by low ‘background’ activity of IP_3_Rs.

Non-excitable cells rely on SOCE as the main [Ca^2+^]_ER_-reloading mechanism for efficient calcium signalling mediated by IP_3_Rs. To activate SOCE, STIM1 has to translocate to the ER–PM junctions [44]. Indeed the presence of STIM1-competent ER–PM junctions in the proximity of the leading edge has recently been reported [7,33]. We therefore next investigated the relative positioning of IP_3_R1, STIM1 and ER–PM junctions.

### Repositioning of IP_3_Rs from cell–cell contacts to the leading edge of migrating cells is accompanied by the accumulation of ER–PM junctions and STIM1 puncta in the adjacent cytoplasmic region

**Figure 4:**
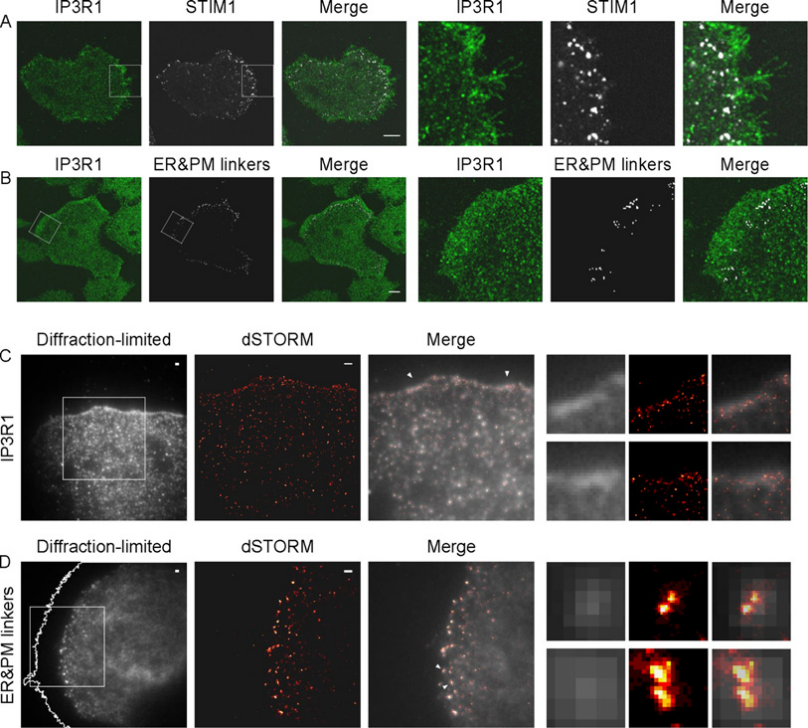
Relative positioning of IP_3_R1 and STIM1/ER–PM junctions in migrating PANC-1 cells (**A**) In migrating PANC-1 cells, IP_3_R1s decorate the leading edge, whereas STIM1 puncta concentrate in the adjacent region (just behind the leading edge). PANC-1 cells were transfected with TK–YFP–STIM1 and then treated with 30 μM CPA to reveal STIM1 puncta. Cells were then fixed and immunostained using antibodies against IP_3_R1. All images in (**A**) and (**B**) show confocal sections taken from ventral parts of the cells located in the immediate proximity to the coverslip. Scale bars represent 10 μm. (**B**) In migrating PANC-1 cells IP_3_R1s decorate the leading edge, whereas ER–PM junctions concentrate in the adjacent region (behind the leading edge). PANC-1 cells were simultaneously transfected with linker constructs ER-targeted CFP–FRB–LL and PM-targeted LL–FKBP–mRFP. Cells expressing both linkers were treated with 100 nM rapamycin to reveal ER–PM junctions. Cells were then fixed and immunostained using antibodies against IP_3_R1. It is informative to compare the observed relative positioning of ER–PM junctions and IP_3_R1 in migrating cells with that in cellular clusters. We found that on confocal sections closest to the coverslip ER–PM junctions were preferentially localized at the cell periphery. Interestingly, some ER–PM junctions were found just behind the IP_3_R1s that decorated cell–cell contacts (Supplementary Fig-ure S5). (**C**) Super-resolution microscopy of IP_3_R1s at the leading edge of a PANC-1 cell. Left panel: the leading edge of a cell immunostained using antibodies against IP_3_R1s and imaged using a TIRF microscope (here and in **D** ‘diffraction-limited’ refers to its lateral resolution). Scale bar represents 1 μm. The fragment, highlighted as a square in the left panel, was then imaged using dSTORM and the result is shown in the central panel. Scale bar represents 1 μm. Right panel (Merge): co-positioning of the two images. Expanded fragments in the right part of (**C**) (small panels) are taken from the peripheral regions indicated by arrowheads in the ‘Merge’ (image left arrowhead corresponds to the upper set of images). (**D**) Super-resolution microscopy of ER–PM junctions near the leading edge of PANC-1 cells. PANC-1 cells simultaneously transfected with both linker constructs (PM-targeted FBKP–LL–mRFP and ER-targeted FRB–LL–CFP) were fixed after treatment with 100 nM rapamycin to highlight the pre-existing ER–PM junctions without ER Ca^2+^ store depletion. PANC-1 cells were then stained using anti-GFP antibody (which also recognizes CFP) to reveal ER-targeted FRB–LL–CFP accumulated in ER–PM junctions. Left panel: the localization of ER–PM junctions visualized using TIRF mode. Scale bar represents 1 μm. The PM border outline was generated using the Threshold and Wand (tracing) tool function of ImageJ (see Supplementary Figure S6). The fragment, highlighted as a square in the left panel, was then imaged using dSTORM and the result is shown in the central panel. Scale bar represents 1 μm. Right panel (Merge): the co-positioning of the two images. Expanded fragments in the right part of (**D**) (small panels) are taken from the peripheral regions highlighted in the Merge image by arrowheads (upper arrowhead corresponds to the upper set of images). Note the improvement of resolution in comparison with diffraction-limited images (**C** and **D**, lateral dSTORM resolution in these experiments was approximately 60–70 nm).

In migrating PANC-1 cells, the region with the increased density of IP_3_R1s at the leading edge was closely followed by the region with a high density of STIM1 puncta (Figure 4A). Similar relative positioning was seen for IP_3_R1s and co-localized ER and PM linkers (white dots highlighting the ER–PM junctions in Figure 4B), suggesting that the ER–PM junctions migrating just behind the IP_3_Rs are STIM/SOCE-competent. The similarity of distributions of STIM1 puncta and ER–PM junctions (identified by the linkers) is consistent with other studies that observed the co-localization of STIM1 with ER–PM junctions [21,33]. The preferential localization of IP_3_R1s and ER–PM junctions at the front of migrating cells was also confirmed using super-resolution microscopy. In these experiments, employing the dSTORM technique, we observed that the leading edge of migrating cells indeed had increased density of IP_3_R1s (Figure 4C) and higher concentration of ER–PM junctions (Figure 4D). Note the increased resolution of dSTORM images (in the *x*–*y* plane) in comparison with diffraction-limited (in the *x*–*y* plane) TIRF images taken from the same cellular regions (insets in Figures 4C and 4D). The actual size of both the ER–PM junctions and clusters of IP_3_Rs is significantly smaller than the limit of resolution of diffraction-limited microscopy but the preferential localization at the leading edge was observed using all types of microscopy. dSTORM imaging, which has considerably improved axial and lateral resolution in comparison with conventional microscopy, confirmed that both IP_3_R1s and ER–PM junctions can be observed close to the leading edge and in the immediate proximity to the ventral membrane of the migrating cells (i.e. portion of the membrane that is involved in forming contacts with the substratum and that is sliding along the substratum). A number of recent studies reported the importance of Ca^2+^ signalling for cell migration and invasion [5–7,45–47]. The Ca^2+^ responses have been shown to both potentiate [7,46] and suppress [7] migration, depending on cell type and extracellular environment. Considering the observed prominent stratified localization of IP_3_Rs and STIM1/ER–PM junctions near the leading edge of migrating PANC-1 cells and the proximity of these structures to the components of migratory apparatus (e.g. focal adhesions and actin fibres) we next decided to test the importance of IP_3_Rs and SOCE for the migration of this cell type.

### Inhibition of IP_3_Rs and STIM–Orai channels suppresses migration of PANC-1 cells

**Figure 5:**
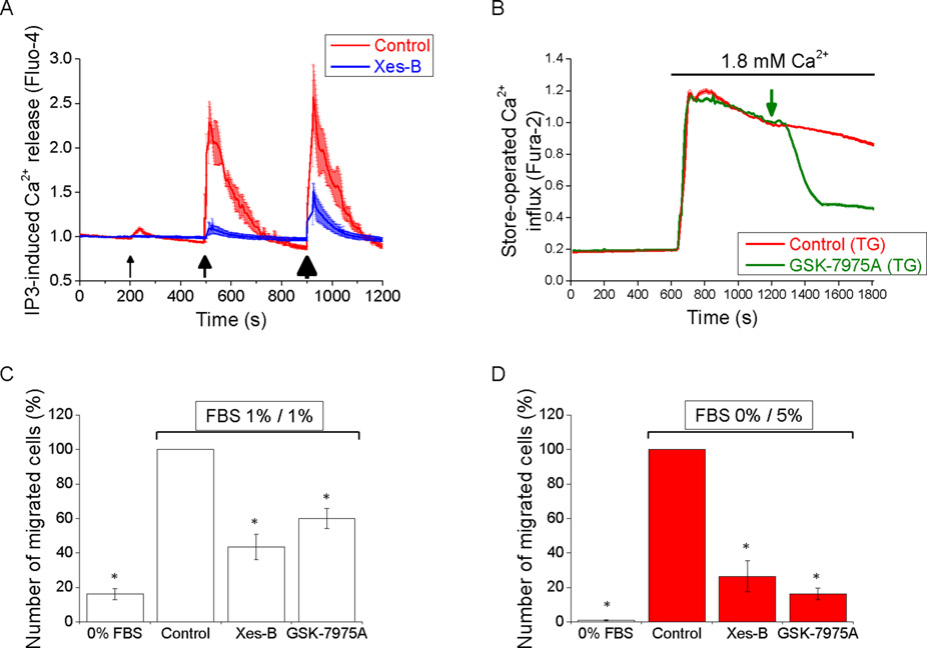
Inhibition of Ca^2+^ signalling complexes suppresses PANC-1 cell migration (**A**) Xestospongin-B (Xes-B) blocks IP_3_-induced Ca^2+^ release in pancreatic adenocarcinoma cells. Ca^2+^ releases from internal stores were measured in cells loaded with caged IP_3_ in the absence (control; red trace, 28 cells) or presence of 50 μM xestospongin B (blue trace, 29 cells). The traces were composed of normalized Fluo-4 fluorescence measurements (mean values ± S.E.M. for individual time points). Pulses of UV light induced the IP_3_ uncaging (i.e. release of IP_3_ from its caged precursor) and subsequent releases of Ca^2+^ from internal stores into the cytosol. The intensities of the black arrows indicate the duration of uncaging (3, 8 and 20 s). In these experiments nominally Ca^2+^-free extracellular solution was used to reveal cytosolic Ca^2+^ responses occurring specifically due to Ca^2+^ release from the intracellular stores. (**B**) GSK-7975A inhibits SOCE in PANC-1 cells. Cells were pre-treated with TG for approximately 20 min before the beginning of the experiments in order to permanently deplete the ER Ca^2+^ stores, and maintained in nominally Ca^2+^-free solution. Increase in extracellular [Ca^2+^] to 1.8 mM resulted in Ca^2+^ influx via the SOCE pathway. Red trace shows control experiment (192 cells). Green trace (151 cells) shows significant inhibition of SOCE by 30 μM GSK-7975A. The green arrow indicates the moment of GSK-7975A addition. The traces were composed from normalized mean values ± S.E.M. for individual time points of Fura-2 fluorescence ratio (*F*340/*F*380) measurements. (**C** and **D**) Xestospongin-B and GSK-7975A suppress migration of PANC-1 cells. PANC-1 cells were subjected to both symmetric (1% FBS in both chambers) (**C**) and asymmetric (0% FBS in the upper chamber and 5% FBS in the lower chamber) (**D**) Boyden chamber migration assay for 6 h at 37°C in the absence (Control) or presence of the inhibitors. 0% FBS conditions (in both chambers, leftmost bars on both panels) were employed for comparison (this effectively inhibits cell migration). The number of migrated cells in each inhibitor-treated group was normalized to that in the control group for every individual experiment. Means ± S.E.M. were obtained from at least three independent experiments. In all cases the number of cells migrated in the presence of an inhibitor was statistically different from that in the control conditions.

**Figure 6:**
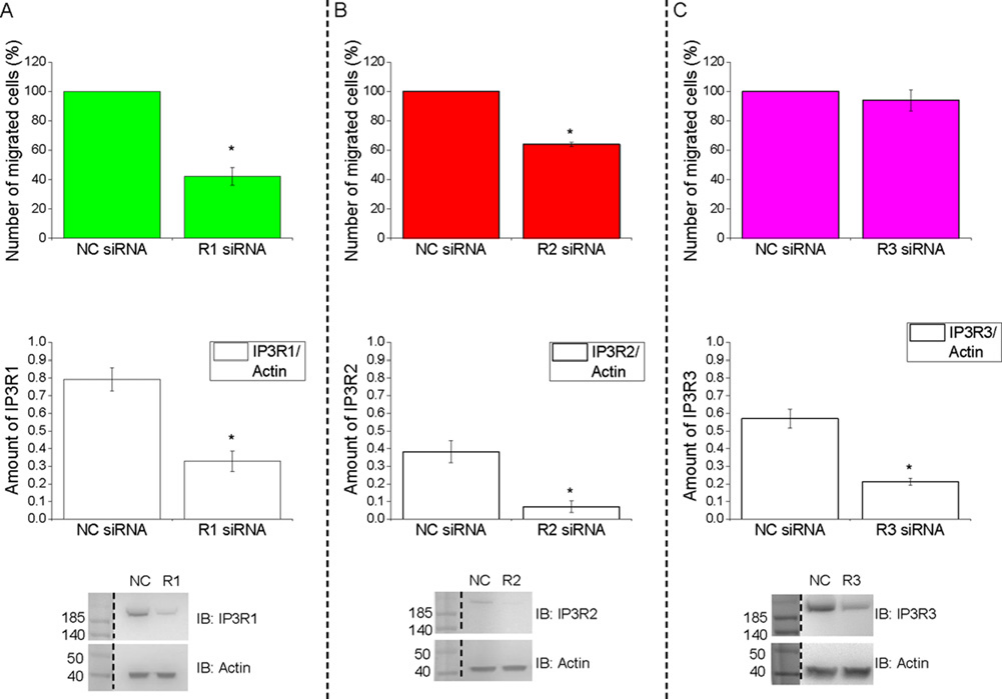
Cellular depletion of IP_3_Rs suppresses pancreatic adenocarcinoma cell migration siRNA knockdown of IP_3_R1 (**A**), IP_3_R2 (**B**) but not IP_3_R3 (**C**) inhibits migration of PANC-1 cells. The cells were treated for 72 h post-transfection with IP_3_R siRNAs (R1 siRNA, R2 siRNA and R3 siRNA) or non-targeting siRNA (NC siRNA) oligomer sequences. After this treatment cell migration was measured using Boyden chamber assay with asymmetric FBS content (0% FBS in the upper chamber and 5% FBS in the lower chamber). In these experiments cells migrated for 6 h at 37°C/5% CO_2_. The number of migrated cells in each siRNA group was normalized to that in the corresponding non-targeting control group. At least three independent experiments were performed for each condition. Western blot profiles showing successful knockdown of IP_3_Rs by siRNA (lower panels). IP_3_Rs and actin protein levels were quantified using the ImageJ densitometry tool. Actin blot was used as a loading control. The amount of IP_3_R proteins was obtained as a function of normalization to the amount of actin protein. At least three independent experiments were performed for each condition. Molecular masses are indicated in kDa. IB, immunoblot.

The selective inhibitor of IP_3_Rs–xestospongin-B [48]–effectively suppressed cytosolic Ca^2+^ responses induced by IP_3_ uncaging in PANC-1 cells (Figure 5A). SOCE in this cell type was significantly (by 61±1%, *n*=151) inhibited by 30 μM GSK-7975A (Figure 5B), a selective inhibitor of SOCE mediated by STIM–Orai interaction [49]. Note that 10 μM GSK-7975A produced only a slightly weaker inhibition than 30 μM (inhibited by 53±1%, *n*=162; results not shown) and 100 μM was not more effective than 30 μM (*n*=145; results not shown). Migration in our experiments was tested using Boyden chambers. In the absence of FBS, PANC-1 cells migrate very inefficiently (leftmost bars in Figures 5C and 5D). We therefore investigated the effect of the inhibitors on migration of these cells in the presence of FBS using symmetrical FBS distribution (1% FBS in both the upper and lower chambers, Figure 5C) and asymmetrical FBS distribution (0% FBS in the upper chamber and 5% FBS in the lower chamber; this configuration can be considered as a model of chemotactic migration, see Figure 5D). Xestospongin-B significantly inhibi-ted migration of PANC-1 cells in conditions of symmetrical FBS (Figure 5C). The effects of this IP_3_R inhibitor on migration were even stronger for cells migrating along the gradient of FBS (Figure 5D); in this condition xestospongin-B inhibited migration by 74±9%. These findings are consistent with the results of IP_3_R-knockdown experiments that suggested the involvement of IP_3_Rs (particularly IP_3_R1 and possibly IP_3_R2) in migration (Figure 6). GSK-7975A supressed migration of PANC-1 cells (Figures 5C and 5D) and, as for xestospongin-B, the effect was particularly prominent in the experiments with asymmetrical FBS (Figure 5D, in these experiments GSK-7975A inhibited migration by 84±3%). Both xestospongin-B and GSK-7975A also inhibited cell migration as measured by wound-healing assay (Supplementary Figure S7). Neither xestospongin-B nor GSK-7975A induced substantial cellular toxicity (Supplementary Figure S8). Strong inhibition of migration by xestospongin-B and GSK-7975A suggest that the striking accumulation of IP_3_Rs and STIM/SOCE-competent ER–PM junctions in the leading edge of PANC-1 cells has a clear function, which is to provide signals important for migration of this type of cancer cells. There was a difference between the effect of xestospongin-B and GSK-7975A on the paxillin content of focal adhesions. Incubation for 1 h with xestospongin-B produced a statistically significant decrease in paxillin content in the focal adhesions of unstimulated PANC-1 cells (see Figure 3C); we did not, however, observe changes in paxillin content in focal adhesions following 1 h of application of 30 μM GSK-7975A (results not shown; *n*=44 for GSK-7975A treated and *n*=39 for the control group). It is therefore conceivable that the two inhibitors utilize different mechanisms for supressing the cell migration and that Ca^2+^ release and influx regulate different processes contributing to the cell migration.

It is interesting to note that in primary normal pancreatic acinar cells IP_3_Rs are preferentially positioned in close proximity to the tight junctions in the cell–cell contact regions near the apical part of the cells [14,50–53]. Indeed, we verified and confirmed this previously reported distribution using the same antibodies as employed for immunostaining of IP_3_Rs in PANC-1 cells (see Supplementary Figure S9). PDAC cells probably originate from pancreatic acinar cells (evidence for this has recently been reviewed in [54]). Therefore the changes in the distribution of IP_3_Rs from cell–cell contacts to the leading edge, that were characterized using a cellular model in our study, are likely to reflect the pathophysiological process associated with EMT *in vivo*. In other words the present study suggests a novel switch of polarity and function of Ca^2+^ signalling complexes during EMT associated with cancerogenesis. Ca^2+^ signalling complexes move from the intercellular contact regions (proximal to the apical part of the cell) to the leading edge and change function from regulating vital physiological processes (exocytosis and fluid secretion) to regulating migration of cancer cells. It is important to note that the leading edge attracts not only IP_3_Rs but also ER–PM junctions and STIM1 and that both Ca^2+^ release and Ca^2+^ influx are important for migration.

The present study describes prominent changes in the distribution of Ca^2+^ signalling complexes that develop when cancer cells disconnect from their neighbours and form a migratory ‘mesenchymal’ phenotype. The observed preferential distribution of IP_3_Rs and STIM1/ER–PM junctions signifies the formation of novel structural and functional (signalling) polarity in migrating PDAC cells. Disrupting this polarized distribution could present a mechanism for inhibiting migration and invasion of PDAC cells and ultimately suppressing the formation of metastasis of this type of cancer.

## Abbreviations

AM: acetoxymethyl ester
BAPTA: 1,2-bis-(*o*-aminophenoxy)ethane-*N,N,N′,N′*-tetra-acetic acid
[Ca^2+^]_C_: Ca^2+^ concentration in the cytosol
[Ca^2+^]_ER_: Ca^2+^ concentration in the ER
CPA: cyclopiazonic acid
EMT: epithelial–mesenchymal transition
ER: endoplasmic reticulum
FKBP: FK506-binding protein
FRP: FKBP12rapamycin-binding
HRP: horseradish peroxidase
IP_3_: inositol trisphosphate
IP_3_R: inositol trisphosphate receptor
LL: long linker
mRFP: monomeric RFP
NA: numerical aperture
Pax-GCaMP5: paxillin labelled with Ca^2+^ sensor GCaMP5
Pax-mCh: mCherry-labelled paxillin
PDAC: pancreatic ductal adenocarcinoma
PFA: paraformaldehyde
PM: plasma membrane
SOCE: store-operated Ca^2+^ entry
STIM: stromal interaction molecule
TG: thapsigargin
TIRF: total internal reflection fluorescence
TK: thymidine kinase

## References

[1] Siegel R., Naishadham D., Jemal A. (2013). Cancer statistics, 2013. CA Cancer J. Clin..

[2] Hanahan D., Weinberg R.A. (2011). Hallmarks of cancer: the next generation. Cell.

[3] Lamouille S., Xu J., Derynck R. (2014). Molecular mechanisms of epithelial-mesenchymal transition. Nat. Rev. Mol. Cell Biol..

[4] Ridley A.J. (2011). Life at the leading edge. Cell.

[5] Evans J.H., Falke J.J. (2007). Ca^2+^ influx is an essential component of the positive-feedback loop that maintains leading-edge structure and activity in macrophages. Proc. Natl. Acad. Sci. U.S.A..

[6] Tsai F.C., Meyer T. (2012). Ca^2+^ pulses control local cycles of lamellipodia retraction and adhesion along the front of migrating cells. Curr. Biol..

[7] Tsai F.C., Seki A., Yang H.W., Hayer A., Carrasco S., Malmersjo S., Meyer T. (2014). A polarized Ca^2+^, diacylglycerol and STIM1 signalling system regulates directed cell migration. Nat. Cell Biol..

[8] Prevarskaya N., Skryma R., Shuba Y. (2011). Calcium in tumour metastasis: new roles for known actors. Nat. Rev. Cancer.

[9] Streb H., Irvine R.F., Berridge M.J., Schulz I. (1983). Release of Ca^2+^ from a nonmitochondrial intracellular store in pancreatic acinar cells by inositol-1,4,5-trisphosphate. Nature.

[10] Berridge M.J. (2009). Inositol trisphosphate and calcium signalling mechanisms. Biochim. Biophys. Acta.

[11] Yule D.I. (2001). Subtype-specific regulation of inositol 1,4,5-trisphosphate receptors: controlling calcium signals in time and space. J. Gen. Physiol..

[12] Hogan P.G., Lewis R.S., Rao A. (2010). Molecular basis of calcium signaling in lymphocytes: STIM and ORAI. Annu. Rev. Immunol..

[13] Parekh A.B., Putney J.W. (2005). Store-operated calcium channels. Physiol. Rev..

[14] Lur G., Haynes L.P., Prior I.A., Gerasimenko O.V., Feske S., Petersen O.H., Burgoyne R.D., Tepikin A.V. (2009). Ribosome-free terminals of rough ER allow formation of STIM1 puncta and segregation of STIM1 from IP(3) receptors. Curr. Biol..

[15] Orci L., Ravazzola M., Le Coadic M., Shen W.W., Demaurex N., Cosson P. (2009). From the Cover: STIM1-induced precortical and cortical subdomains of the endoplasmic reticulum. Proc. Natl. Acad. Sci. U.S.A..

[16] Wu M.M., Buchanan J., Luik R.M., Lewis R.S. (2006). Ca^2+^ store depletion causes STIM1 to accumulate in ER regions closely associated with the plasma membrane. J. Cell Biol..

[17] Lefkimmiatis K., Srikanthan M., Maiellaro I., Moyer M.P., Curci S., Hofer A.M. (2009). Store-operated cyclic AMP signalling mediated by STIM1. Nat. Cell Biol..

[18] Willoughby D., Everett K.L., Halls M.L., Pacheco J., Skroblin P., Vaca L., Klussmann E., Cooper D.M. (2012). Direct binding between Orai1 and AC8 mediates dynamic interplay between Ca^2+^ and cAMP signaling. Sci. Signal..

[19] Chang C.L., Hsieh T.S., Yang T.T., Rothberg K.G., Azizoglu D.B., Volk E., Liao J.C., Liou J. (2013). Feedback regulation of receptor-induced Ca^2+^ signaling mediated by E-Syt1 and Nir2 at endoplasmic reticulum-plasma membrane junctions. Cell Rep..

[20] Kar P., Samanta K., Kramer H., Morris O., Bakowski D., Parekh A.B. (2014). Dynamic assembly of a membrane signaling complex enables selective activation of NFAT by Orai1. Curr. Biol..

[21] Varnai P., Toth B., Toth D.J., Hunyady L., Balla T. (2007). Visualization and manipulation of plasma membrane-endoplasmic reticulum contact sites indicates the presence of additional molecular components within the STIM1-Orai1 complex. J. Biol. Chem..

[22] Feske S., Gwack Y., Prakriya M., Srikanth S., Puppel S.H., Tanasa B., Hogan P.G., Lewis R.S., Daly M., Rao A. (2006). A mutation in Orai1 causes immune deficiency by abrogating CRAC channel function. Nature.

[23] Liou J., Kim M.L., Heo W.D., Jones J.T., Myers J.W., Ferrell J.E., Meyer T. (2005). STIM is a Ca^2+^ sensor essential for Ca^2+^-store-depletion-triggered Ca^2+^ influx. Curr. Biol..

[24] Park C.Y., Hoover P.J., Mullins F.M., Bachhawat P., Covington E.D., Raunser S., Walz T., Garcia K.C., Dolmetsch R.E., Lewis R.S. (2009). STIM1 clusters and activates CRAC channels via direct binding of a cytosolic domain to Orai1. Cell.

[25] Roos J., DiGregorio P.J., Yeromin A.V., Ohlsen K., Lioudyno M., Zhang S., Safrina O., Kozak J.A., Wagner S.L., Cahalan M.D. (2005). STIM1, an essential and conserved component of store-operated Ca^2+^ channel function. J. Cell Biol..

[26] Yuan J.P., Zeng W., Dorwart M.R., Choi Y.J., Worley P.F., Muallem S. (2009). SOAR and the polybasic STIM1 domains gate and regulate Orai channels. Nat. Cell Biol..

[27] Worley P.F., Zeng W., Huang G.N., Yuan J.P., Kim J.Y., Lee M.G., Muallem S. (2007). TRPC channels as STIM1-regulated store-operated channels. Cell Calcium.

[28] Mogami H., Gardner J., Gerasimenko O.V., Camello P., Petersen O.H., Tepikin A.V. (1999). Calcium binding capacity of the cytosol and endoplasmic reticulum of mouse pancreatic acinar cells. J. Physiol..

[29] Zhou Z., Neher E. (1993). Mobile and immobile calcium buffers in bovine adrenal chromaffin cells. J. Physiol..

[30] Dingli F., Parys J.B., Loew D., Saule S., Mery L. (2012). Vimentin and the K-Ras-induced actin-binding protein control inositol-(1,4,5)-trisphosphate receptor redistribution during MDCK cell differentiation. J. Cell Sci..

[31] Akerboom J., Chen T.W., Wardill T.J., Tian L., Marvin J.S., Mutlu S., Calderon N.C., Esposti F., Borghuis B.G., Sun X.R. (2012). Optimization of a GCaMP calcium indicator for neural activity imaging. J. Neurosci..

[32] Laukaitis C.M., Webb D.J., Donais K., Horwitz A.F. (2001). Differential dynamics of alpha 5 integrin, paxillin, and alpha-actinin during formation and disassembly of adhesions in migrating cells. J. Cell Biol..

[33] Dingsdale H., Okeke E., Awais M., Haynes L., Criddle D.N., Sutton R., Tepikin A.V. (2013). Saltatory formation, sliding and dissolution of ER-PM junctions in migrating cancer cells. Biochem. J..

[34] Burdyga A., Conant A., Haynes L., Zhang J., Jalink K., Sutton R., Neoptolemos J., Costello E., Tepikin A. (2013). cAMP inhibits migration, ruffling and paxillin accumulation in focal adhesions of pancreatic ductal adenocarcinoma cells: effects of PKA and EPAC. Biochim. Biophys. Acta.

[35] Metcalf D.J., Edwards R., Kumarswami N., Knight A.E. (2013). Test samples for optimizing STORM super-resolution microscopy. J. Vis. Exp..

[36] Ferraro F., Kriston-Vizi J., Metcalf D.J., Martin-Martin B., Freeman J., Burden J.J., Westmoreland D., Dyer C.E., Knight A.E., Ketteler R., Cutler D.F. (2014). A two-tier Golgi-based control of organelle size underpins the functional plasticity of endothelial cells. Dev. Cell.

[37] Rees E., Erdelyi M., Pinotsi D., Knight A.E., Metcalf D., Kaminski C.F. (2012). Blind assessment of localisation microscope image resolution. Optical Nanoscopy.

[38] Rees E.J., Miklos Erdelyi M., Kaminski Schierle G.S., Knight A., Kaminski C.F. (2013). Elements of image processing in localization microscopy. J. Optics.

[39] Colosetti P., Tunwell R.E., Cruttwell C., Arsanto J.P., Mauger J.P., Cassio D. (2003). The type 3 inositol 1,4,5-trisphosphate receptor is concentrated at the tight junction level in polarized MDCK cells. J. Cell Sci..

[40] Horiguchi K., Shirakihara T., Nakano A., Imamura T., Miyazono K., Saitoh M. (2009). Role of Ras signaling in the induction of snail by transforming growth factor-beta. J. Biol. Chem..

[41] Nakajima S., Doi R., Toyoda E., Tsuji S., Wada M., Koizumi M., Tulachan S.S., Ito D., Kami K., Mori T. (2004). N-cadherin expression and epithelial-mesenchymal transition in pancreatic carcinoma. Clin. Cancer Res..

[42] Deakin N.O., Turner C.E. (2008). Paxillin comes of age. J. Cell Sci..

[43] Kim D.H., Wirtz D. (2013). Focal adhesion size uniquely predicts cell migration. FASEB J..

[44] Carrasco S., Meyer T. (2011). STIM proteins and the endoplasmic reticulum-plasma membrane junctions. Annu. Rev. Biochem..

[45] Nicol X., Hong K.P., Spitzer N.C. (2011). Spatial and temporal second messenger codes for growth cone turning. Proc. Natl. Acad. Sci. U.S.A..

[46] Wei C., Wang X., Chen M., Ouyang K., Song L.S., Cheng H. (2009). Calcium flickers steer cell migration. Nature.

[47] Motiani R.K., Hyzinski-Garcia M.C., Zhang X.X., Henkel M.M., Abdullaev I.F., Kuo Y.H., Matrougui K., Mongin A.A., Trebak M. (2013). STIM1 and Orai1 mediate CRAC channel activity and are essential for human glioblastoma invasion. Pflugers Arch..

[48] Jaimovich E., Mattei C., Liberona J.L., Cardenas C., Estrada M., Barbier J., Debitus C., Laurent D., Molgó J. (2005). Xestospongin B, a competitive inhibitor of IP3-mediated Ca^2+^ signalling in cultured rat myotubes, isolated myonuclei, and neuroblastoma (NG108–15) cells. FEBS Lett..

[49] Derler I., Schindl R., Fritsch R., Heftberger P., Riedl M.C., Begg M., House D., Romanin C. (2013). The action of selective CRAC channel blockers is affected by the Orai pore geometry. Cell Calcium.

[50] Lee M.G., Xu X., Zeng W.Z., Diaz J., Wojcikiewicz R.J. H., Kuo T.H., Wuytack F., Racymaekers L., Muallem S. (1997). Polarized expression of Ca^2+^ channels in pancreatic and salivary gland cells–correlation with initiation and propagation of [Ca^2+^](i) waves. J. Biol. Chem..

[51] Nathanson M.H., Fallon M.B., Padfield P.J., Maranto A.R. (1994). Localization of the type-3 inositol 1,4,5-trisphosphate receptor in the Ca^2+^ wave trigger zone of pancreatic acinar-cells. J. Biol. Chem..

[52] Yule D.I., Ernst S.A., Ohnishi H., Wojcikiewicz R.J. H. (1997). Evidence that zymogen granules are not a physiologically relevant calcium pool–defining the distribution of inositol 1,4,5-trisphosphate receptors in pancreatic acinar cells. J. Biol. Chem..

[53] Lur G., Sherwood M.W., Ebisui E., Haynes L., Feske S., Sutton R., Burgoyne R.D., Mikoshiba K., Petersen O.H., Tepikin A.V. (2011). InsP(3) receptors and Orai channels in pancreatic acinar cells: co-localization and its consequences. Biochem. J..

[54] Rooman I., Real F.X. (2012). Pancreatic ductal adenocarcinoma and acinar cells: a matter of differentiation and development?. Gut.

